# Protective effects of swimming exercises and metformin on cardiac and aortic damage caused by a high-fat diet in obese rats with type 2 diabetes, by regulating the Bcl2/Bax signaling pathway

**DOI:** 10.55730/1300-0144.5727

**Published:** 2023-08-11

**Authors:** Ebru ÖZÜDOĞRU, Emrah ATAY, Mehtap SAVRAN, Halil AŞCI, Özlem ÖZMEN, Şenay TOPSAKAL

**Affiliations:** 1 Burdur Mehmet Akif Ersoy University, Institute of Education Sciences, Department of Physical Education and Sports Education, Burdur, Turkiye; 2 Burdur Mehmet Akif Ersoy University, Faculty of Sport Science, Department of Physical Education and Sport, Burdur, Turkiye; 3 Süleyman Demirel University, Faculty of Medicine, Department of Pharmacology, Isparta, Turkiye; 4 Burdur Mehmet Akif Ersoy University, Faculty of Veterinary Medicine, Department of Pathology, Burdur, Turkiye; 5 Pamukkale University, Faculty of Medicine, Department of Endocrinology and Metabolism, Denizli, Turkiye

**Keywords:** Bcl2, diabetes mellitus, heart, inflammation, nesfatin-1, swimming exercise

## Abstract

**Background/aim:**

Due to the increasing mortality and morbidity rates in diabetes mellitus (DM), which is one of the biggest health problems of our age, many treatment modalities are still being tried. The positive effects of metformin (MET) and physical exercise (EXE) on the pathophysiology of diabetes are well known. In this study, it was aimed to detail these positive effects of MET and EXE in combination on the basis of inflammation, apoptosis mechanisms, and endogen nesfatin-1 (NES-1) synthesis.

**Materials and methods:**

Twenty-seven type 2 DM (DM-2) male Wistar Albino rats were divided into 4 groups, as the high-fat diet (HFD), MET, EXE, and MET+EXE groups. The total duration of the study was 3 months. At the end of the experiment, blood glucose and lipid profiles were measured. Histopathological evaluation was performed on the cardiac and aortic tissues and apoptotic markers were evaluated immunohistochemically. Inflammatory markers and NES-1 levels were analyzed by enzyme-linked immunosorbent assay.

**Results:**

The plasma glucose, homeostatic model evaluation-insulin resistance (HOMA-IR), low-density lipoprotein (LDL) levels increased, and high-density lipoprotein (HDL) levels decreased significantly in the HFD group. In the treatment groups, the glucose, HOMA-IR, LDL, NES-1 levels in the plasma, as well as tumor necrosis factor alpha (TNF-α), interleukin-1 beta (IL-1β), IL-6, caspase-3 (Cas-3), Bcl-2-associated X protein (Bax), and histopathological findings of inflammation in tissues were decreased. Additionally, there was an increase in plasma insulin, HDL, and tissue B-cell lymphoma-2 and levels.

**Conclusion:**

It was observed that the MET and EXE treatments in the DM-2 model reduced cellular damage mechanisms such as inflammation and apoptosis. The decrease in NES-1 levels was thought to be secondary to this antiinflammatory effect. In conclusion, the results demonstrated the effectiveness of EXE in reducing DM-2 and the NES-1 levels. Further studies are needed to evaluate the effect in different EXE models and treatment durations.

## 1. Introduction

Currently, obesity is one of the biggest health problems, leading to various subsequent conditions such as heart attack, diabetes, hypertension, cancer, and even premature death [1]. Many factors such as excess energy intake, insufficient energy consumption, low fat oxidation, and decreased physical activity play a role in the progression of obesity caused by a high-fat diet (HFD) [2]. These factors basically affect the control mechanisms that regulate energy input and output, causing the energy balance to shift towards intake. The positive amount of energy resulting from the disruption of the energy balance leads to an increase in adipose tissue. As a result, an abnormal increase in body weight occurs [3].

Obesity is an important risk factor in the development of diabetes mellitus (DM) and is a metabolic disorder that often accompanies type 2 DM (DM-2). Although the relationship between obesity and DM-2 remains unclear, tumor necrosis factor alpha (TNF-α) levels in adipocytes, and insulin and receptor dysfunction are thought to be some of the underlying mechanisms [4]. DM-related hyperlipidemia and hyperglycemia play a key role in the development of damage in blood and tissues by causing mitochondrial stress [5]. The B-cell lymphoma-2 (Bcl2)/Bcl-2-associated X protein (Bax) ratio is an indicator of mitochondrial activity in cardiac myocytes and regulates apoptosis [6]. Therefore, it is important in elucidating the degree of mitochondrial damage and the mechanism of protective effects.

Nesfatin-1 (NES-1) is a neurohormone mainly secreted from the hypothalamus and synthesized from nucleobindin-2, known as the satiety peptide, and has effects on the appetite mechanism and nutritional status [7]. It is closely associated with weight loss, malnutrition, and glucose homeostasis [8]. This new hormone can be used as an agent in the early diagnosis of obesity or obesity-related metabolic disorders.

The blood glucose-lowering effect of metformin (MET) occurs primarily by the inhibition of gluconeogenesis in the liver. It also inhibits glycogenolysis, increases insulin-mediated glucose uptake in skeletal muscle and adipocytes, and decreases intestinal glucose absorption [9]. MET exerts all of these effects by affecting the signaling mechanism that regulates energy metabolism [10,11].

Planned and programmed exercise (EXE) has a significant effect on maintaining body weight control and preventing obesity. Increased muscle activity during EXE plays a vital role in the energy balance by increasing the activation of metabolic systems and accelerating energy expenditure. Regulation of the energy metabolism is one of the most remarkable benefits of EXE in diseases characterized by metabolic disorders such as obesity and diabetes [12,13].

Different results have been reported in studies aiming to show the relationship between EXE and NES-1 [14–16]. Additionally, there are a limited number of published studies on the effects of MET on NES-1 in obese populations with DM-2. It is noteworthy that there are no studies on the effect of regular EXE with or without MET use on the NES-1 hormone in obese rats with DM-2.

Thus, the aim of this study was to investigate the protective effect of regular EXE and MET on cardiovascular damage in obese rats with DM-2, in terms of the NES-1 levels and Bcl-2/Bax ratio.

## 2. Materials and methods

### 2.1. Ethical approval for animal studies

All of the experiments were performed in accordance with the Animal Research: Reporting in Vivo Experiments (ARRIVE) Guidelines 2.0, while the experimental study protocol was approved by the Animal Experiments Local Ethics Committee of Burdur Mehmet Akif Ersoy University (MAKU-HADYEK) (Ethics No: 17.02.2021/720). Animals were obtained from the Burdur Mehmet Akif Ersoy University Experimental Animal Production and Experimental Research Center. The rats were housed in rat rooms with 1 rat in each Euro type-2 cage under constant temperature (22 ± 2 °C) and humidity (55%–60%) conditions and a 12:12 day/night photoperiod.

### 2.2. HFD content and the induction of obesity

Rats weighing 220 ± 20 g were obtained from the experimental animal center and fed a HFD to induce obesity (>300 g of body weight). All of the rats were fed a HFD (30.82% crude fat, 17.73% crude protein, 18.46% crude fiber, and 5.16% crude ash) ad libitum, which was prepared by the researcher, until the end of the experiment. Standard rat feed was ground into powder in a mill, and then 500 g of melted beef tallow was added to each 1000 g of powdered rat feed [17], which was mixed until homogenized. Warm water was added to the mixture to make a dough, which was then formed into pellets in refrigerator bags and baked at 180° for 20 min. The high-fat feed prepared by the researcher was analyzed at the Laboratory of Animal Nutrition and Nutrition Diseases Department of Burdur Mehmet Akif Ersoy University (Faculty of Veterinary Medicine) with registration number 21-027. The ratios of standard pellet feed and high-fat feed are given in Table 1. All of the animals were fed this feed for 4 weeks and then rats that weighed 300 g or more were considered obese.

### 2.3. Induction of DM-2

Streptozotocin (STZ; Cayman Chemical, Ann Arbor, MI, USA) was administered intraperitoneally (i.p.) at a dose of 35 mg/kg to rats that reached the target weight for obesity to create a DM-2 model [18]. Two days after STZ administration, only 20 of 32 rats could be included in the study, since their glucose levels were higher than 200 mg/dL according to the results of blood samples taken from the tail vein. STZ was readministrated to these 12 rats. Two days later, 7 of these STZ-treated rats had glucose levels of 200 mg/dL or above and were also included in the study. A total of 27 obese and diabetic rats were thus included in the study. DM-2 was controlled with glucose measurements at 15-day intervals.

### 2.4. Study groups

Twenty-seven male, 3–6-month-old, Wistar Albino rats, with obesity and DM-2, were divided into 4 groups, as follows:

1. HFD group (n = 6): No treatment other than being fed a HFD (control group).
2. MET group (n = 7): Fed a HFD and the daily administration of MET (Glifor 1000 mg film tablet; Bilim İlaç, İstanbul, Türkiye), added to the drinking water at a dose of 100 mg/kg throughout the experiment [19].
3. EXE group (n = 7): Fed a HFD and performing regular swimming exercises (EXEs).
4. EXE+MET group (n = 7): Fed a HFD feeding, performing regular swimming EXEs, and the administration of MET (conditions the same as the MET group).

During the experiment, glucose levels in the blood taken from the tail vein were checked twice a month. In addition, weekly weight monitoring of the animals was performed for 12 weeks. One day after the last EXE and drug administration, under xylazine hydrochloride (10 mg/kg) (Alfazin, Alfasan IBV) and ketamine hydrochloride (90 mg/kg) (Alfamin, Alfasan International B.V., Netherlands) anesthesia, the abdomens of the rats were opened, and blood samples were collected from the inferior vena cava. The rats were euthanized by surgical exsanguination. The blood samples were centrifuged at a low speed and plasma was separated for biochemical analysis and the serum glucose, high-density lipoprotein (HDL), low-density lipoprotein (LDL), and insulin levels were analyzed. The NES-1 expression and inflammation parameters such as interleukin-6 (IL-6), interleukin-1 beta (IL-1β), and TNF-α were examined using enzyme-linked immunosorbent assay (ELISA). Heart and aortic samples were fixed in 10% buffered formaldehyde solution for the histopathological and immunohistochemical evaluation of caspase-3 (Cas-3), Bax, and Bcl-2 after blood sampling.

### 2.5. Exercise protocol

**Figure 1:**
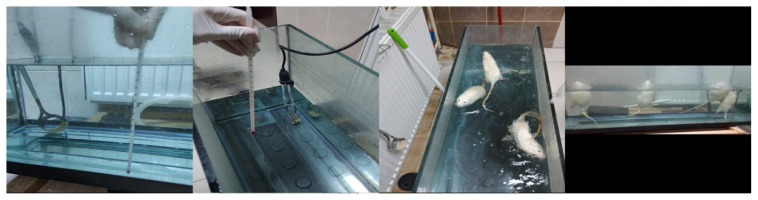
The rats performing swimming EXEs.

A swimming pool made of thick glass material that was at least 20–25 cm long, 40–50 cm deep, and 80–100 cm wide was prepared for the swimming EXEs (Figure 1).

Before starting the study, swimming training was given to all of the rats to ensure that the rats could swim in the pool without stress. The water level in the pool was adjusted so that their noses remained above the water when their hind legs touched the bottom of the pool to prevent them from being stressed. The water temperature during the EXE was maintained at 23 °C using a water thermostat. The temperature and hygiene of the water was checked every EXE day (Figure 1).

The EXE and EXE+MET groups underwent 30 min of swimming EXEs, 3 days a week. The EXEs lasted for a total of 8 weeks. The time required for the swimming training was not included in this period.

### 2.6. Biochemical analysis

#### 2.6.1. Analysis of the blood samples

At the end of the study, blood samples taken during the necropsy of the rats were examined for biochemical analysis at the Burdur Mehmet Akif Ersoy University Veterinary Faculty Animal Hospital Laboratory. The HDL, LDL, and glucose contents in the serum were analyzed spectrophotometrically with Randox chemical kits using a Randox Monaco RX5002 analyzer (Randox Laboratories Ltd., Crumlin, County Antrim, UK).

#### 2.6.2. Measurement of tissue NES-1, IL-1β, IL-6, and TNF-α levels

Quantitative measurement of the NES-1 levels in the heart and aortic tissue and the IL-6, IL-1β, and TNF-α levels in the heart tissue were performed by competitive ELISA using a commercial kit (BT Lab, Shanghai Korain Biotech Co., Ltd., Shanghai, China). The kits included 6-point calibrators. The standard concentrations were 75, 150, 300, 600, 1200, and 2400 ng/L for NES-1; 300 , 600 , 1200 , 2400 , 4800 , and 9600 pg/mL for IL-1β; 1.5, 3, 6, 12, 24, and 48 ng/L for IL-6; and 40, 80, 160, 320, 640, and 1280 ng/L for TNF-α. All of standards were run in duplicate and optical density data corresponding to these concentrations were obtained. At the end of the procedure, the optical density-concentration graph of the standards was plotted, and the concentrations of all of the samples were calculated using this graph. Measurement sensitivities were 5.24 ng/L for NES-1, 10.23 pg/mL for IL-1β, 0.052 ng/L for IL-6, and 2.51 ng/L for TNF-α. For all of the parameters, the intra-assay coefficient of variation (CV) was <8%, and the inter-assay CV was <10%. Protein concentrations of all of the studied tissues were measured, and all of the parameters were given as concentrations per gram of tissue.

#### 2.6.3. Measurement of the serum insulin levels

Quantitative measurement of the venous blood insulin level was performed by competitive ELISA using a commercial kit (BT Lab). The kit contained 6-point calibrators (1.5, 3, 6, 12, 24, and 48 mIU/L) and all of the standards were run in duplicate. Optical density data corresponding to these concentrations were obtained. At the end of the procedure, the optical density-concentration graph of the standards was drawn, and the concentrations of all of the samples were calculated in mIU/L using this graph. The sensitivity was 0.05 mIU/L. For all of the parameters, the intra-assay CV was <8% and the inter-assay CV was <10%.

#### 2.6.4. Calculation of insulin resistance

Homeostatic model evaluation-insulin resistance (HOMA-IR) was used to calculate the insulin resistance of the rats. The HOMA-IR values were calculated as (HOMA-IR = fasting serum insulin level (μU/ML) × fasting serum glucose level (mmol/L) /22.5) [20].

### 2.7. Histopathological analysis

At the end of the study, all of the rats were euthanized and necropsied. Cardiac and aortic tissue samples collected during the necropsy were immediately placed in a 10% buffered formalin solution for fixation. The following day, the specimens were trimmed, transferred to tissue processing cassettes, and kept in the formalin solution for one more day to complete the fixation process. During the trimming process, utmost care was taken to collect all of the heart samples from the same area. The samples were then placed in a fully automatic tissue processing device (Leica ASP300S; Leica Microsystems, Wetzlar, Germany) and subjected to routine procedures. The samples were then embedded in paraffin wax. Sections measuring 5 μm thick were placed on a fully automatic rotary microtome (Leica 2155, Leica Microsystems) and stained with Harris hematoxylin and eosin (H&E). Finally, the slides were coverslipped and evaluated under a light microscope. Microphotographs were taken to reflect the appearance of the study groups. Myocardial tissue and endothelial cell damage were assessed using a semiquantitative scoring scale of 0–5, modified from that of Zhang et al. [21]. The scoring was performed as follows: 0 = normal myocardial and endothelial cells; 1 = a scant number of myocardial cells (<5%) showing necrosis or endothelial sloughing (1 or 2 cells/high magnification); 2 = 5%–15% of myocardial cells showing necrosis or endothelial sloughing; 3 = multiple foci of necrotic myocardial cells (16%–25%) and slight inflammatory cell infiltration at the myocardium or 16%–25% of endothelial cells lost; 4 = myocardial cells (26%–35%) showing necrosis and moderate inflammatory cell infiltration or 26%–35% of endothelial cells lost; 5 = more than 35% of myocardial cells showing necrosis, severe inflammatory cell infiltration, and more than 35% of endothelial cells lost.

### 2.8. Immunohistochemical analysis

Three separate serial sections were taken from cardiac and aortic tissues, drawn on poly-L-lysine slides, and stained according to the streptavidin-biotin complex peroxidase method. Bcl-2 (Bcl-2 antibody, A11434, AFG Bioscience, Northbrook, IL, USA, 1/100 dilution), Bax (Bax antibody, A11427, AFG Bioscience, 1/50 dilution), Cas-3 (caspase-3 (anticaspase-3 antibody (ab4051), Abcam Cambridge, UK, 1/100 dilution) staining was completed according to the immunohistochemical staining procedure and the manufacturers’ instructions. The sections were incubated with primary antibody for 60 min before incubation with biotinylated secondary antibody and streptavidin-alkaline phosphatase conjugate. As a secondary antibody, the EXPOSE Mouse and Rabbit Specific HRP/DAB Detection IHC kit (ab80436, Abcam) was used. Diaminobenzidine (DAB) was used as a chromogen. Instead of primary antibody, antigen dilution solution was used as a negative control. All of the examinations were carried out on blinded samples. Immunohistochemical expressions were scored semiquantitatively from 0 to 3, with 0 = negative, 1 = mild, 2 = moderate, and 3 = strongly positive. Database Manual Cell Sens Life Science Imaging Software System (Olympus Co., Tokyo, Japan) was used for the morphometric analysis and microphotography. Immunohistochemical scores and cell counting analysis were performed using ImageJ version 1.48 (National Institutes of Health, Bethesda MD). Statistical analysis was carried out on the scores and differences between the study groups were determined.

### 2.9. Statistical analysis

IBM SPSS Statistics for Windows 22.0 (IBM Corp., Armonk, NY, USA) was used for the statistical analysis. One-way analysis of variance (ANOVA) was used for comparisons between independent groups. The homogeneity of variance assumption was also checked with this test, and when the homogeneity of variance was ensured, the least significant difference (LSD) post hoc test was used for pairwise comparisons. The margin of error for all of the tests was < 0.05.

## 3. Results

### 3.1. Biochemical analysis

The blood glucose levels in the HFD group were significantly decreased in comparison with the MET, EXE, and MET+EXE groups (p < 0.001 for all). In addition, the glucose levels in the MET group were significantly less than in the EXE group (p < 0.05). A significant increase in the insulin levels was observed in the MET group compared to the HFD and EXE groups (p < 0.01 for both). The HOMA-IR values were significantly lower in the MET, EXE, and MET+EXE groups compared to the HFD group (p < 0.05 for all). When the effect of the MET and EXE on changes in the lipid profile was examined, it was determined that the HDL levels increased significantly in the plasma of the MET, EXE, and MET+EXE groups compared to the HFD group (p < = 0.01, p < 0.05, and p < 0.01, respectively). When the treatment groups were compared with each other, there was no significance between the groups (p > 0.05). Plasma LDL levels significantly increased in the HFD group, whereas this increment was reversed significantly in the MET (p < 0.01), EXE (p < 0.01), and MET+EXE (p < 0.001) groups. The LDL levels in the MET+EXE group were lower than those in both the MET and EXE groups (p < 0.001 for both) (Table 2).

### 3.2. NES-1 and inflammation markers in heart and aorta tissue

When the NES-1 levels in the aortic tissue were examined, no statistical significance was found between the groups (p > 0.05). The cardiac NES-1 levels were significantly increased in the HFD group, and it was significantly lower in the other groups (p ≤ 0.001 for all). There was no statistical difference between the treatment groups (p > 0.05) (Table 3).

In the HFD group, the TNF-α, IL-1β, and IL-6 levels were also increased significantly (p < 0.05), while all 3 treatment groups exhibited significantly reversed changes (p < 0.05 for the MET group, p < 0.01 for the EXE group, and p < 0.01 for the MET+EXE group). When the comparison between the treatment groups was made, no statistical significance was found for any of the markers (p > 0.05) (Table 3).

### 3.3. Histopathological analysis

**Figure 2:**
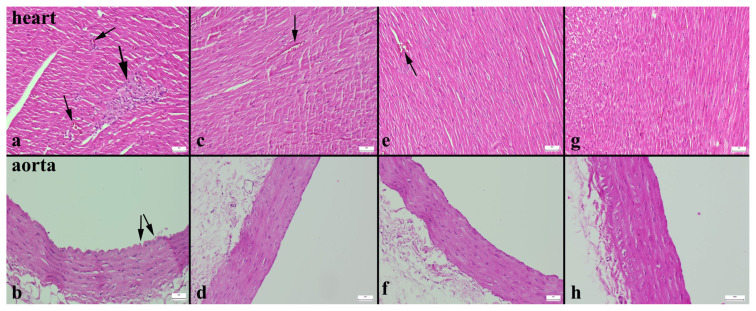
Histopathological evaluations of the cardiac and aortic tissues of the rats. Hyperemia (thin arrows) and inflammatory cell infiltrations (thick arrows) in the myocardial vessels (a) and shedding of endothelial cells (b) in the HFD group. Inflammatory reaction and significantly reduced hyperemia (arrow) (c) and normal aorta histology (d) in the MET group. Significantly reduced hyperemia (arrow) (e) and normal aorta (g) in the EXE group. Completely normal histology for both tissues in the MET+EXE group (g, h). Results were statistically significant compared the control group (<0.001 for all), H&E, bars = 50 μm.

In the histopathological examination of the heart and aortic tissues, severe hyperemia, small hemorrhage in the vessels of the myocardium, and mild inflammatory cell infiltrations in some areas with shedding vascular endothelia were noted in the HFD group (Figures 2a and 2b). These findings decreased significantly in the other groups (Figures 2c–2h), while the most significant decrease was observed in the MET+EXE group (Figures 2g and 2h). No marked pathological findings were detected in the aortas of the rats, except in the HFD group. Statistical analysis results of the histopathological scores are shown in Table 4.

### 3.4. Immunohistochemical findings

**Figure 3:**
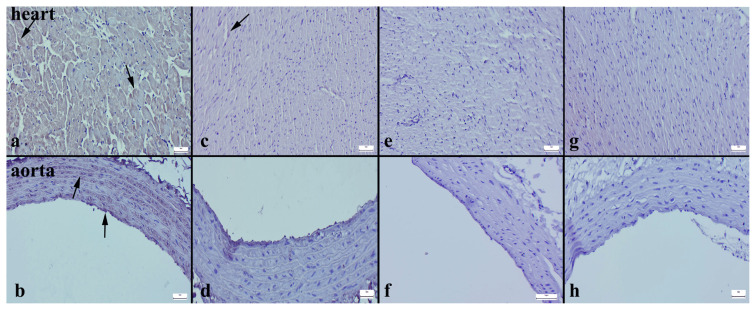
Cas-3 immunoexpressions of the cardiac and aortic tissues of the rats. Increased Cas-3 immunoreaction (arrows) in the myocardium and aorta in the HFD group (a, b). Significantly decreased Cas-3 expressions in both tissues for all of the other groups (c–h). Streptavidin biotin peroxidase method, bars = 50 μm.

**Figure 4:**
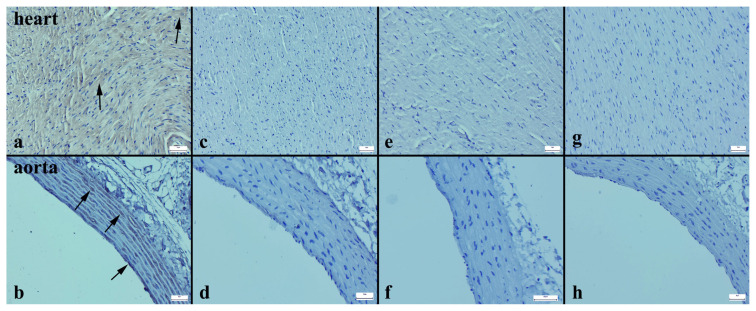
Bax immunoexpressions of the cardiac and aortic tissues of the rats. Increased Bax immunoreaction (arrows) in the myocardium and aorta in the HFD group (a, b). Significantly decreased Bax expressions in both tissues for all of the other groups (c–h). Streptavidin biotin peroxidase method, bars = 50 μm.

**Figure 5:**
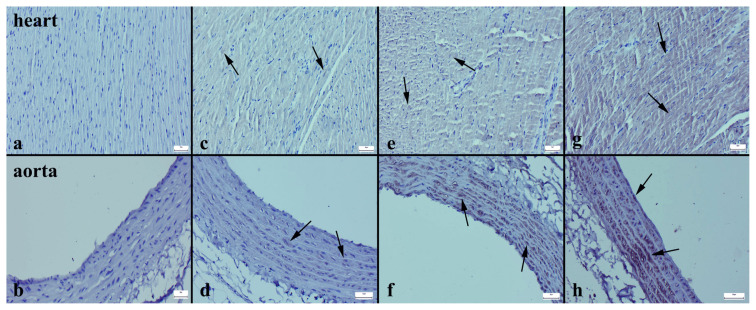
Bcl-2 immunoexpressions of the cardiac and aortic tissues of the rats. Negative Bcl-2 immunoreaction (arrows) in the myocardium and aorta in the HFD group (a, b). Significantly increased Bcl-2 expressions in both tissues for all of the other groups (c–h). Streptavidin biotin peroxidase method, bars = 50 μm.

The Cas-3 expression in both the heart and aortic tissues significantly increased in the HFD group (Figures 3a and 3b), while in the other groups, there was a noticeable decrease (Figures 3c–3h). Similarly, when examining the Bax immunohistochemical expression, there was a substantial increase in both tissues in the HFD group (Figures 4a and 4b), whereas in the other groups, a decrease was evident (Figures 4c–4h). In contrast, while the Bcl-2 immunoreaction significantly decreased in the HFD group (Figures 5a and 5b), the expression increased in all of the treatment groups (Figures 5c–5h) (p < 0.001 for all) (Table 4).

## 4. Discussion

DM-2 is a disease with high morbidity and mortality, which is very common worldwide and progresses with multiorgan damage [22,23]. DM-2 progresses with hyperinsulinemia secondary to hyperglycemia and resistance develops over time due to the continuous stimulation of insulin receptors, especially in patients with a BMI >30 [24,25]. As a result of this resistance, despite the increased insulin levels in the blood, glucose levels cannot be reduced, and the energy source required for the cells cannot be provided. This can have detrimental effects on organelles such as mitochondria and may even result in cellular death [6].

The cardiovascular system is one of the most important structures to be affected by tissue damage due to DM and a HFD. Hemostasis mechanisms may result in endothelial damage and increased lipid molecules may further exacerbate this situation [26].

In this study, the high glucose and HOMA-IR levels observed in the HFD group showed that the disease model was well established. The reason for the low insulin levels in the HFD group may be considered as a reflection of STZ-induced pancreatic beta cell damage. The decreased insulin levels in the HFD group indicated beta cell dysfunction or a decreased reserve. It was expected that the glucose and HOMA-IR levels would be lower with MET administration, which decreases insulin resistance and increases the use of glucose at tissue level. This drug, which is the most commonly used oral agent in routine practice, had a positive effect on the insulin requirement of rats fed a HFD due to the changes in the insulin levels by inhibiting beta cell damage [27]. The decrease in the blood glucose and HOMA-IR levels observed in the EXE group is an indication that EXE increased the cellular glucose uptake, but the lack of an increase in the insulin levels indicates that EXE did not have a positive effect on beta cell destruction. The increased insulin levels in the EXE+MET group may be interpreted as an indication that MET can improve beta cell damage due to its antioxidant, antiinflammatory, and antiapoptotic properties.

DM-related hyperglycemia is usually associated with hyperlipidemia, which may cause atherosclerosis and related organ damage [28,29]. In this study, the levels of HDL, which transport fat molecules to the liver to break it down, increased in the MET and EXE groups, proving that both approaches prevent the development of atherosclerotic conditions. One of the most important outcomes of this study is that this treatment caused a decrease in the LDL levels. EXE and MET were able to reduce the levels of LDL, which carry blood cholesterol, and thus make an important contribution to the prevention of cardiovascular pathologies in terms of lipid regulation. Consistent with these results, Zong et al. obtained similar HDL and LDL results in their DM-2 rat model [30]. Furthermore, Baretti et al. showed that obese rat LDL levels decreased by 61% with EXE [31].

Oxidative stress and inflammation are also triggered by glycosylation products that develop in the blood due to DM-2 [32–34]. It is known that these 2 pathological events, which are among the basic cell damage mechanisms, trigger intracellular pathways and the synthesis of various inflammatory cytokines including TNF-α, IL-1β, and IL-6 from the cell nucleus. The role of TNF-α in this inflammatory scenario is also related to insulin resistance [35,36]. In the current study, the decreased inflammatory cytokine levels in the treatment groups are an indication of the regression of the pathological process occurring in the HFD group.

The antiinflammatory efficacy of EXE and/or MET treatment is consistent with the histopathological and immunostaining results. Similar to the results herein, Kim et al. reported that increased TNF-α, IL-1β, and IL-6 levels in the blood decreased with EXE in STZ-induced DM rats [37].

Apoptosis occurs by both the external pathway in which inflammation-mediated TNF-α plays key role, and the internal pathway, which is mainly regulated by mitochondrial damage [38]. EXE, especially through its positive effects on oxygen metabolism, decreased mitochondrial stress and slowed down the development of apoptosis via the intrinsic pathway. Decreased Bax and Cas-3, and increased Bcl-2 levels in the treatment groups herein support this. In the study of Habibi et al., the Bcl-2 levels decreased and Bax levels increased in the heart tissue of rats with DM [39]. These changes were reversed by EXE.

NES-1 is an anorexigenic peptide that increases insulin secretion from beta islet cells, reduces damage in some disease models, and contributes to antioxidant, antiinflammatory, and antiapoptotic effects [40]. The fact that no significant difference was observed between the NES-1 levels of the aortic tissues in the current study suggests that NES-1 is not as effective in endothelial protection as it is thought to be. The minimal change in the NES-1 levels is a secondary contribution to the reduction of inflammation with drugs and EXE, although not directly in endothelial protection. Feijoo-Bandín et al. found that the NES-1 levels varied according to diet and coronary health [41]. They also showed that cardiomyocytes can synthesize NES-1, which can induce glucose uptake and the mobilization of glucose transporter-4. Naseroleslami et al. demonstrated that NES-1 showed antiinflammatory activity by suppressing the expression of proinflammatory cytokines [42]. They also determined that NES-1 showed antiapoptotic activity by inhibiting Cas-3. In this study, it can be interpreted that the regression in inflammation, oxidative stress, and apoptosis caused by MET and EXE decreased the expression of NES-1.

In conclusion, in the current study, it was observed that the use of MET in combination with EXE in DM rats fed a HFD ameliorated cardiovascular disease risk conditions, but NES-1 was not directly effective, or its levels decreased secondary to a reduction in tissue damage. However, further studies with different EXE programs or durations are needed.

## Figures and Tables

**Table 1:** Standard pellet feed and high fat feed ratios.

Standard pellet feed		High fat feed	
Raw protein	24.00%	Raw protein	17.73%
Raw cellulose	5.96%	Raw cellulose	18.46%
Raw oil	5.88%	Raw oil	30.82%
Ash	5.37%	Ash	5.16%

**Table 2:** Plasma glucose and lipid profiles

	HFD	MET	EXE	MET- EXE	p-value
Glucose	632.04 ± 79.76^a^	359.03 ± 71.77^b^	456.05 ± 73.47^c^	418.70 ± 52.81^b,c^	<0.05
Insulin	4.55 ± 0.33^a^	6.08 ± 0.29 ^b^	4.81 ± 1.01^a^	5.27 ± 1.09^a,b^	<0.01
HOMA-IR	7.11 ± 1.10^a^	5.37 ± 0.99^b^	5.41 ± 1.45^b^	5.51 ± 1.53^b^	<0.05
HDL	11.00 ± 2.36^a^	15.00 ± 2.38^b^	14.42 ± 3.40^b^	15.14 ± 1.86^b^	<0.05
LDL	23.33 ± 2.92^a^	18.50 ± 4.44^b^	19.14 ± 2.34^b^	12.00 ± 2.51^c^	<0.05

HFD: high-fat diet, MET: metformin, EXE: exercise, HOMA-IR: homeostatic model assessment of insulin resistance, HDL: high density lipoprotein, LDL: low density lipoprotein. The differences between the means of groups with different letters between the groups are statistically significant. One-way ANOVA, post hoc LSD test.

**Table 3:** NES-1 and inflammation indicators in the heart and aorta tissue.

	HFD	MET	EXE	MET+EXE	p-value
Vascular NES-1	102.60 ± 31.61^a^	80.41 ± 28.15^a^	94.40 ± 12.36^a^	79.86 ± 22.36^a^	>0.05
Cardiac NES-1	117.02 ± 14.10^a^	87.44 ± 18.98^b^	75.31 ± 11.81^b^	76.64 ± 7.42^b^	≤0.001
Cardiac TNF-α	57.68 ± 15.61^a^	44.53 ± 14.64^b^	39.31 ± 2.39^b^	40.61 ± 4.60^b^	<0.05
Cardiac IL-1β	421.01 ± 89.25^a^	311.63 ± 60.92^b^	271.03 ± 73.20^b^	269.95 ± 66.71^b^	<0.05
Cardiac IL-6	3.45 ± 0.91^a^	1.64 ± 0.67^b^	1.42 ± 0.18^b^	1.53 ± 0.27^b^	≤0.001

NES-1: nesfatin-1, IL-1β: interleukin-1 beta. Differences between the means of groups with different letters between the groups are statistically significant. One-way ANOVA, post hoc LSD test.

**Table 4:** Statistical analysis results of the immunohistochemical scores for the groups.

	HFD	MET	EXE	MET + EXE	p-value
Histo-heart	2.83 ± 0.75^a^	0.57 ± 0.20^b^	0.28 ± 0.18^b^	0.42 ± 0.20^b^	<0.001
Histo-aort	2.33 ± 0.51^a^	0.42 ± 0.20^b^	0.42 ± 0.20^b^	0.28 ± 0.18^b^	<0.001
Cas-3 heart	2.33 ± 0.51^a^	0.42 ± 0.20^b^	0.28 ± 0.18^b^	0.14 ± 0.14^b^	<0.001
Cas-3 aort	1.66 ± 0.81^a^	0.28 ± 0.18^b^	0.28 ± 0.18^b^	0.14 ± 0.14^b^	<0.001
Bax heart	2.50 ± 0.54^a^	0.57 ± 0.29^b^	0.42 ± 0.20^b^	0.14 ± 0.14^b^	<0.001
Bax aort	2.33 ± 0.81^a^	0.57 ± 0.53^b^	0.28 ± 0.18^b^	0.14 ± 0.14^b^	<0.001
Bcl-2 heart	0.33 ± 0.21^a^	1.85 ± 0.69^b^	2.00 ± 0.81^b^	2.57 ± 0.53^b^	<0.001
Bcl-2 aort	0.16 ± 0.16^a^	2.14 ± 0.37^bc^	1.71 ± 0.48^c^	2.42 ± 0.53^d^	<0.001

Cas-3: caspase-3. Differences between the means of groups with different letters between the groups are statistically significant. One-way ANOVA, post hoc LSD test.
